# LBD29-Involved Auxin Signaling Represses NAC Master Regulators and Fiber Wall Biosynthesis^1^ ^[OPEN]^

**DOI:** 10.1104/pp.19.00148

**Published:** 2019-08-03

**Authors:** Kwang-Hee Lee, Qian Du, Chunliu Zhuo, Liying Qi, Huanzhong Wang

**Affiliations:** a Department of Plant Science and Landscape Architecture, University of Connecticut, Storrs, Connecticut 06269; b Department of Biological Sciences, University of North Texas, Denton, Texas 76203; c Institute for Systems Genomics, University of Connecticut, Storrs, Connecticut 06269

## Abstract

NAM, ATAF1/2 and CUC2 (NAC) domain transcription factors function as master switches in regulating secondary cell wall (SCW) biosynthesis in Arabidopsis (*Arabidopsis thaliana*) stems. Despite the importance of these NACs in fiber development, the upstream signal is still elusive. Using a large-scale mutant screening, we identified a dominant activation-tagging mutant, *fiberless-d* (*fls-d*), showing defective SCW development in stem fibers, similar to that of the *nac secondary wall thickening promoting factor1-1* (*nst1-1*)*nst3-3* double mutant. Overexpression of *LATERAL ORGAN BOUNDARIES DOMAIN29* (*LBD29*) is responsible for the *fls-d* mutant phenotypes. By contrast, loss-of-function of *LBD29*, either in the dominant repression transgenic lines or in the transfer-DNA (T-DNA) insertion mutant *lbd29-1*, enhanced SCW development in fibers. Genetic analysis and transgenic studies demonstrated *LBD29* depends on master regulators in mediating SCW biosynthesis, specifically *NAC SECONDARY WALL THICKENING PROMOTING FACTOR1* (*NST1*), *NST*2, and *NST*3. Increasing indole-3-acetic acid (IAA) levels, either in stem tissues above a *N*-1-naphthylphthalamic acid–treated region or in plants directly sprayed with IAA, inhibits fiber wall thickening. The inhibition effect of naphthylphthalamic acid treatment and exogenous IAA application depends on a known auxin signaling pathway involving AUXIN RESPONSE FACTOR7 (ARF7)/ARF19 and LBD29. These results demonstrate auxin is upstream of LBD29 in repressing NAC master regulators, and therefore shed new light on the regulation of SCW biosynthesis in Arabidopsis.

## Teaser

The NAC domain master regulators of secondary cell wall biosynthesis are negatively regulated by auxin signaling through a pathway that involves ARF7/ARF19 and LBD29 in Arabidopsis stem fibers.

Plant fibers develop secondary cell walls (SCWs) that provide mechanical strength for upright growth and represent a major source of plant biomass (Růžička et al., 2015; Zhong and Ye, 2015; Yang and Wang, 2016). The formation of SCW is regulated by a complex transcriptional regulatory network, in which NAM, ATAF1/2, and CUC2 (NAC) domain transcription factors serve as master regulators (Wang and Dixon, 2012; Taylor-Teeples et al., 2015; Yang and Wang, 2016). In Arabidopsis (*Arabidopsis thaliana*) stem fibers, the master regulators of SCW biosynthesis are NAC SECONDARY WALL THICKENING PROMOTING FACTOR1 (NST1), NST2, and NST3/SECONDARY WALL-ASSOCIATED NAC DOMAIN PROTEIN 1 (SND1), whereas the simultaneous loss-of-function of NST1 and NST3 results in the absence of fiber SCW formation (Mitsuda et al., 2007; Zhong et al., 2007; Zhong and Ye, 2015). Downstream of these NACs are numerous other transcription factors that collectively control the expression of SCW biosynthesis genes (Zhong and Ye, 2014; Růžička et al., 2015; Taylor-Teeples et al., 2015; Yang and Wang, 2016). Despite the comprehensive understanding of NACs and their downstream genes, the upstream signal of the NAC master switches in fiber development is still unknown.

Auxin plays important roles in many aspects of plant growth and development, including cambium activity and secondary growth (Lavy and Estelle, 2016; Leyser, 2017). Auxin acts as a positional signal in vein-forming procambial cells in leaves and has a positive role in cambium proliferation in Arabidopsis stems (Uggla et al., 1996; Berleth et al., 2000; Donner et al., 2009; Suer et al., 2011). Perturbation in auxin signaling by reducing responsiveness hinders cambial cell division and restricts fiber and vessel growth (Nilsson et al., 2008). Auxin has been proposed as the ‘body weight’ signal that induces secondary growth in stem tissues (Ko et al., 2004). Besides auxin signaling, auxin transport may have a role in wall thickening. The *interfascicular fiberless 1* (*ifl1*) mutant shows a reduced basipetal auxin transport and fails to form interfascicular fibers (Zhong and Ye, 1999, 2001). Interestingly, treatment of wild-type plants with the polar auxin transport inhibitor naphthylphthalamic acid (NPA) enhances fiber development in the middle part of stems but has no definitive effect at the lower part (Zhong and Ye, 1999, 2001). Mutation of *Walls Are Thin* 1 (*WAT1*), which encodes a plant-specific tonoplast-localized auxin transporter, results in thinner walls in interfascicular fibers (Ranocha et al., 2010). Local application of the synthetic auxin 2, 4-d or naphthylacetic acid restores wall thickness to a wild-type level (Ranocha et al., 2013). Overall, these studies indicate auxin transport may play a positive role in wall biosynthesis. The PIN-FORMED 1 (PIN1) protein is the founding member of auxin efflux carriers that control many aspects of plant development (Estelle, 1998). Surprisingly, no obvious defects in fiber differentiation are observed in *pin1* and other auxin transport-related mutants, indicating the function of auxin transport in fiber wall biosynthesis is still uncertain (Chen et al., 1998; Estelle, 1998; Gälweiler et al., 1998). In contrast with the aforementioned studies, there are also indications auxin may function as a negative regulator of SCW formation. The Vascular-related NAC domain 6 (VND6) is a master regulator for meta-xylem development (Kubo et al., 2005). Exogenous application of auxin alone inhibited *VND6* expression and meta-xylem development in vitro (Kubo et al., 2005; Didi et al., 2015). Physiological and genetic studies suggest auxin could be a negative regulator of cell lignification (Cecchetti et al., 2013; Didi et al., 2015). In tree species, auxin distributes in a radial concentration gradient, with highest levels in the cambial zone, which then decline over the cell expansion regions and are lowest in SCW-forming cells (Uggla et al., 1996; Tuominen et al., 1997). This negative correlation indicates auxin may not be a positive signal for SCW formation.

LATERAL ORGAN BOUNDARIES DOMAIN (LBD) transcription factor proteins that feature a conserved LATERAL ORGAN BOUNDARIES (LOB) domain are key regulators of plant development (Husbands et al., 2007; Xu et al., 2016). The LBD genes *AtLBD16*, *AtLBD17*, *AtLBD18*, and *AtLBD29* are auxin inducible and function downstream of AUXIN RESPONSE FACTOR7 (ARF7) and ARF19 in auxin signaling (Okushima et al., 2007). These LBD proteins regulate several developmental processes, including lateral root formation, callus induction, and adventitious rooting from wounded or detached plant tissues (Okushima et al., 2005, 2007; Fan et al., 2012; Liu et al., 2014). The functions of these four LBDs have diversified, although they may participate in a common developmental process. In response to auxin, LBD29 maintains division capability of the pericycle cells (Feng et al., 2012). In contrast, LBD18 has a role in the specification, as well as subsequent emergence of lateral roots (Lee et al., 2009). In vascular vessel cells, LBD18 may form a positive feedback loop with VND7 that positively regulates tracheary element differentiation (Soyano et al., 2008).

In this study, we report LBD29 is involved in aspects of auxin signaling that inhibits fiber wall thickening in Arabidopsis stems. Increasing auxin levels, in regions above NPA treatment or in plants exposed to an IAA application, negatively affect fiber wall thickening. Previous studies have shown ARF7/ARF19 and their downstream LBDs function in lateral root formation, callus formation, and adventitious rooting (Okushima et al., 2005, 2007; Fan et al., 2012; Liu et al., 2014). This auxin signaling pathway is also involved in fiber wall development. We report SCW development was absent in fibers in an activation-tagging line, *fiberless-d* (*fls-d*), in which *LBD29* expression is activated. In contrast, enhanced fiber wall development was observed in the *LBD29* loss-of-function mutant *lbd29-1* or in transgenic lines expressing a dominant negative construct, *LBD29-SRDX*. The NAC domain master regulators of SCW biosynthesis genes function downstream of LBD29. Our results indicate auxin induces the expression of *LBD29*, which in turn represses SCW biosynthesis in stem fibers. We further discuss a role for auxin function in balancing cambium proliferation and cell differentiation in vascular and interfascicular fibers in Arabidopsis stems.

## RESULTS

### Identification of a Dominant *fls-d* Mutant with Defects in Fiber Wall Development

**Figure 1.**
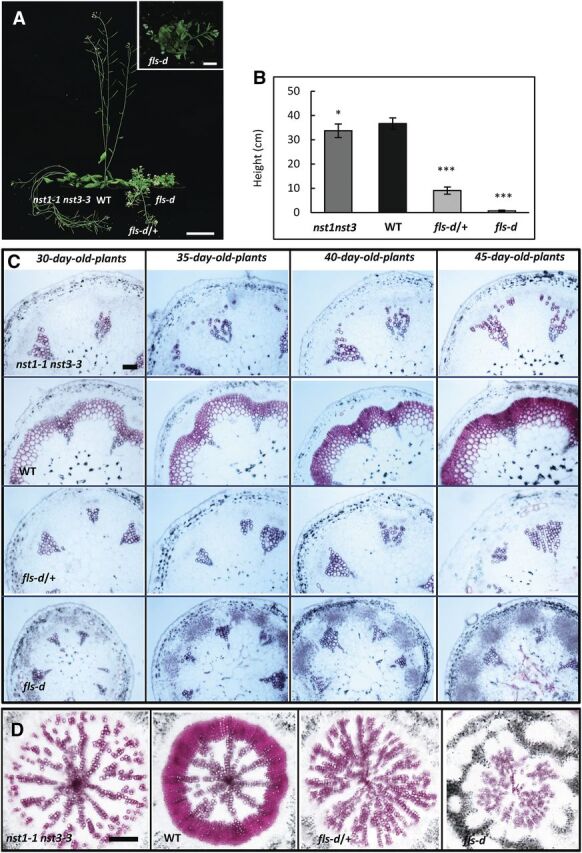
A dominant mutant *fls-d* showing defects in secondary cell wall thickening in fiber cells. A, Plant growth phenotypes of *nst1-1nst3-3* double mutant, wild-type (WT), *fls-d*/+, and *fls-d* mutant plants. The inset is the magnified *fls-d* plant. The *fls-d* mutant plants were pendulous similar to the *nst1-1nst3-3* double mutants. Scale bars = 5 cm. B, Plant height of wild type and mutants. *Significant difference (*P* < 0.05); ***extremely significant difference (*P* < 0.001), Student's *t* test. C, Histochemical characterization of stem cross sections with phloroglucinol staining, indicated by pink color. All sections were prepared from the bottom of the stem just above the rosette leaves. Scale bar = 100 μm. D, Phloroglucinol staining of hypocotyls of *nst1-1nst3-3*, wild-type, and *fls-d* mutant plants. Scale bar = 200 μm.

Mutation of two NAC master regulators of SCW development resulted in a striking fiberless phenotype (Fig. 1, A–C). The stems of the *nst1-1nst3-3* double mutant grew pendulously due to the lack of lignified secondary walls in both vascular and interfascicular regions (Fig. 1, A and C). To gain new insight into the regulation of NAC master switches, we performed a forward genetic screen by taking advantage of a large activation-tagging population in Arabidopsis (Du et al., 2015). Stem cross sections of the mutant plants were prepared and examined under UV fluorescence microscope. Cells with secondary walls, such as vascular vessels and fibers, can be easily detected due to auto-florescence of lignin. We have successfully identified several secondary wall development–related mutants using this approach (Du et al., 2015). We report here a semidominant mutant, *fls-d*, that showed a phenotype with no SCWs in the fascicular and interfascicular fibers, similar to the *nst1-1nst3-3* double mutant.

Compared with wild-type plants, the heterozygous (*fls-d*/+) and homozygous (*fls-d*) mutant plants were dwarf (Fig. 1, A and B). Although the bolting and flowering time were similar, the homozygous *fls-d* plants grew to a height less than one tenth of the wild-type plants (Fig. 1B). In addition, stems of both *fls-d*/+ and *fls-d* plants were pendulous in growth habit, indicating compromised physical strength in stems (Fig. 1A). Wrinkled leaves, small flowers, and short siliques were also observed in the mutant plants (Fig. 1A). To better characterize the development of vascular and interfascicular tissues, we analyzed stem cross sections using histochemical analysis with samples harvested at different stages of maturation, ranging from 30- to 45-d-old plants (Fig. 1C). All stem sections were cut from the bottom of the stem at the level of rosette leaves. Phloroglucinol specifically stains cells with SCWs to a red color. In the wild-type plants, fiber cells accumulate more and more lignified wall materials as evidenced by increased phloroglucinol staining intensity in both the vascular and interfascicular regions (Fig. 1C). In contrast, the *fls-d*/+ and *fls-d* plants showed no staining in fiber cells in both fascicular and interfascicular regions, indicating defective secondary wall development (Fig. 1C). The vascular vessel cells of the mutant lines showed no significant difference from wild-type plants (Fig. 1C). The observed fiberless phenotype of *fls-d* mutant lines was similar to those of the *nst1-1 nst3-3* double mutant (Mitsuda et al., 2007; Zhong et al., 2007). However, the *fls-d* mutant plants were extremely dwarf, whereas *nst1 nst3* plants were only slightly shorter than the wild-type plants. The difference in plant growth indicates the *FLS* gene may have broader functions than specifically regulating fiber wall development.

Hypocotyls produce vascular tissues and are used to study SCW development in Arabidopsis. During primary growth, only vessels develop secondary walls in the xylem region. During secondary growth, both vessels and xylary fibers form thick secondary walls. As expected, the wild-type plants showed strong staining of vessels and xylary fibers in secondary xylem, whereas only vessel cells were observed in the central primary xylem region (Fig. 1D). In contrast, the *nst1-1 nst3-3* double mutant plants developed no SCW in xylary fibers in the secondary xylem area (Fig. 1D). Similarly, we did not observe secondary wall development in xylary fibers in both *fls-d*/+ and *fls-d* mutant lines (Fig. 1D). These results clearly showed that SCW formation in fibers was defective in *fls-d* mutant plants.

### Overexpression of *LBD29* Is Responsible for the *fls-d* Fiberless Phenotype

**Figure 2.**
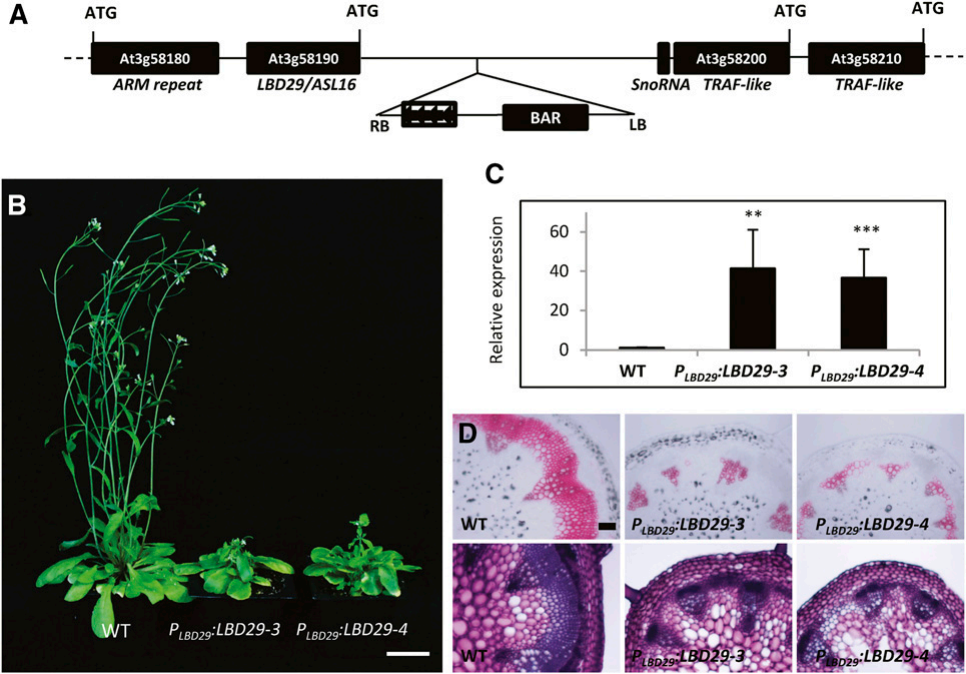
Overexpression of *LBD29* is responsible for the FLS phenotypes. A, Schematic diagram showing the insertion of an activation-tag in the genome of *fls-d*. The insertion is located in the intergenic region upstream of the *LBD29* gene. The *Cauliflower mosaic virus* (CaMV) 35S tetrad enhancer and Bialaphos Resistance (BAR) gene selection marker are marked on the T-DNA. LB, left border; RB, right border. B, Growth phenotypes of wild-type (WT) and transgenic lines expressing a *Pro_LBD29_:LBD29* construct, in which *LBD29* expression was driven by its endogenous promoter. Scale bar = 2.5 cm. C, RT-qPCR analysis of *LBD29* expression in wild-type and transgenic lines. **Significant difference, *P* < 0.01; ***significant difference, *P* < 0.001; Student's *t* test. D, Histochemical characterization of stem cross sections of the *Pro_LBD29_:LBD29* transgenic lines. Top rows are phloroglucinol staining, indicated by pink color, and bottom rows are toluidine blue staining of wild type and two *Pro_LBD29_:LBD29* transgenic lines. Scale bar = 100 μm.

Using a thermal asymmetric interlaced PCR (TAIL-PCR) approach and subsequent sequencing, we identified the activation tag located between *At3G58190* (*LBD29/ASL16*) and *At3G58200* (a *Tumor necrosis factor Receptor-Associated Factor* [*TRAF*]-like gene) in the *fls-d* mutant genome (Fig. 2A). Genotyping of a segregating *fls-d/+* progeny population indicated this insertion was linked with the *fls-d* mutant phenotypes. We then analyzed the expression of the five genes in a 10-KB range from the activation tag using reverse transcription-quantitative PCR (RT-qPCR). Transcription of *LBD29* was up-regulated about 20 times in heterozygous plants and 40 times in homozygous plants, whereas the other four genes were also up-regulated 1.5 to 4 times compared with the wild-type plants (Supplemental Fig. S1).

To investigate the gene(s) responsible for the *fls-d* mutant phenotype, we overexpressed these five genes individually in the wild-type background. Two strategies were used to express the *LBD29* gene. First, we transformed wild-type plants with a construct in which the *LBD29* gene was driven by its endogenous promoter (Fig. 2, B–D). A number of the resulting transgenic plants had much shorter stems compared with wild type (Fig. 2B). RT-qPCR analysis indicated that the *LBD29* gene was overexpressed by about 40-fold in two representative transgenic lines (Fig. 2C). Histochemical analysis of stem cross sections indicated fibers in fascicular and interfascicular regions did not develop SCWs, as shown by phloroglucinol and toluidine blue staining (Fig. 2D). Second, overexpression of the *LBD29* gene was achieved using a CaMV 35S promoter. Similarly, the representative transgenic lines showed dwarf phenotypes (Supplemental Fig. S2A). In addition, the main stems of transgenic plants were unable to grow upright (Supplemental Fig. S2A). When compared with wild-type plants, the *LBD29* gene was overexpressed by about 60-fold in two representative transgenic lines as shown by RT-qPCR analysis (Supplemental Fig. S2B). The development of SCW in fascicular and interfascicular fiber cells was significantly repressed, although the phenotypes were not as strong as the transgenic lines in which *LBD29* overexpression was under its endogenous promoter (Supplemental Fig. S2C). We also overexpressed the other four genes individually in wild-type background but did not observe any defects in either plant growth or fiber development (Supplemental Fig. S1, B–D). These results indicate overexpression of *LBD29* was responsible for the fiberless phenotype observed in *fls-d* mutants. In addition, expression specificity of *LBD29* is important for its function as suggested by phenotypic difference in fiber development in the two overexpression experiments.

### Repression of LBD29 Function or Disruption of *LBD29* Expression Enhances Fiber Wall Development

**Figure 3.**
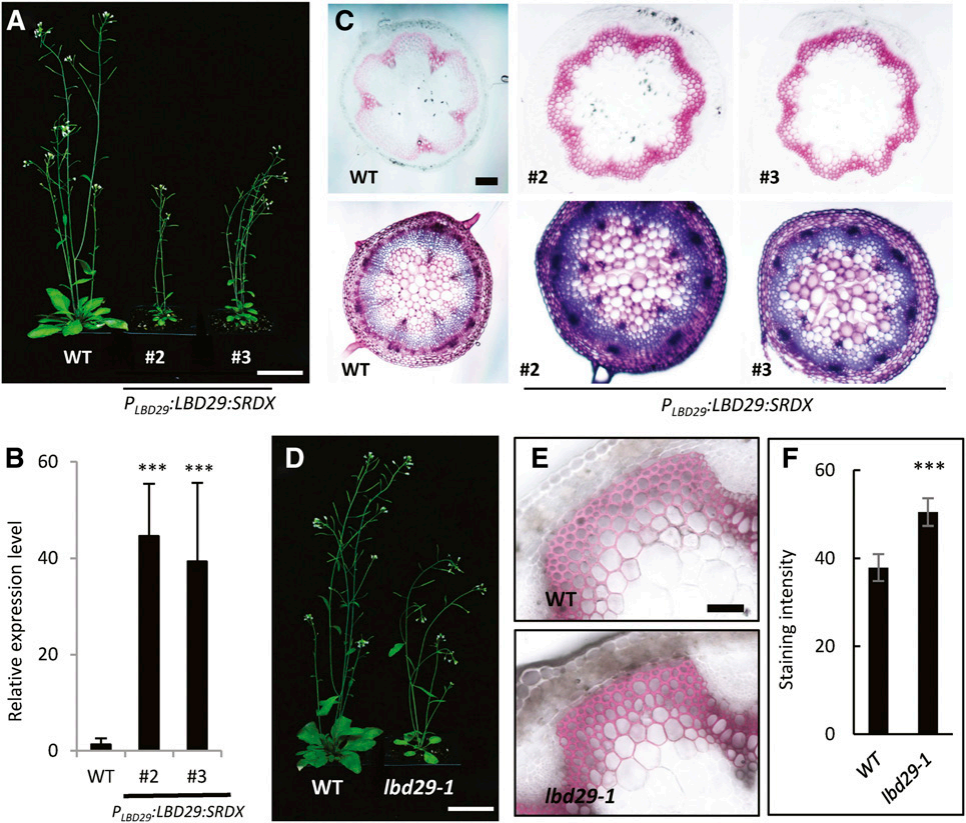
Repression of *LBD29* enhances secondary cell wall development in interfascicular fibers. A, Plant growth phenotypes of wild-type (WT) and two representative transgenic plants expressing a *Pro_LBD29_:LBD29-SRDX* construct (#2 and #3). SRDX is a synthetic motif that confers a dominant negative repression on target genes of LBD29. Scale bar = 5 cm. B, RT-qPCR analysis of *LBD29-SRDX* expression compared with wild type. C, Histochemical characterization of stem cross sections with phloroglucinol staining, indicated by pink color (top), and toluidine blue staining (bottom). Scale bar = 200 μm. D, Plant growth phenotypes of wild type and *lbd29-1*. Scale bar = 5 cm. E, Histochemical characterization of wild-type and *lbd29-1* plants with phloroglucinol staining, indicated by pink color. Scale bar = 50 μm. F, Quantification of the staining intensity of wild-type and *lbd29-1* plants. Fluorescence signal intensity was measured using Image J. ***Significant difference, *P* < 0.001; Student's *t* test.

If overexpression of *LBD29* represses SCW development in fiber cells, we reasoned knocking-down the expression of *LBD29* or interference with its expression pattern may have an opposite effect on wall development. To test our hypothesis, we fused LBD29 with a dominant repression domain, SRDX, which would dominantly repress the transcription of the target genes of LBD29 (Hiratsu et al., 2003). First, we transformed wild-type plants with a *LBD29-SRDX* construct driven by its endogenous promoter (*ProLBD29:LBD29-SRDX*). The stems of the transgenic plants were shorter compared with wild-type plants (Fig. 3A). RT-qPCR analysis indicated the transcripts of *LBD29-SRDX* were highly expressed in the representative transgenic lines (Fig. 3B). When compared with wild-type plants, histochemical staining with phloroglucinol and toluidine blue showed stronger intensity over a broader area in the interfascicular fiber region in transgenic lines, indicating enhanced development of fiber secondary walls (Fig. 3C). These results indicated repression of target genes of LBD29 enhanced cell wall development in fiber cells. Second, we transformed wild-type plants with a *LBD29-SRDX* construct under the control of a CaMV 35S promoter (Supplemental Fig. S3). The transgenic plants were extremely dwarf compared with the wild type (Supplemental Fig. S3A). Expression of the transcripts of *LBD29-SRDX* in these transgenic lines was detected with RT-qPCR analysis (Supplemental Fig. S3B). Histochemical staining showed secondary walls were ectopically developed in phloem cap fiber cells, and even the base of stem trichomes (Supplemental Fig. S3C). These results further support the hypothesis that LBD29 functions as a negative regulator of wall biosynthesis.

In a previous study, a mutant line *lbd29-1* harboring a T-DNA insertion in the promoter region of *LBD29* reduced lateral root formation (Feng et al., 2012). Stems of the *lbd29-1* mutants were a little shorter compared with wild type (Fig. 3D). Staining of stem cross sections with phloroglucinol showed wall development was enhanced in fascicular and interfascicular fiber cells in the *lbd29-1* mutant plants (Fig. 3, E and F). Consistent with the quantified difference in staining, measurements using the acetyl bromide approach showed a significant increase in total lignin content in the *lbd29-1* mutant plants (Supplemental Fig. S4A). This result also indicates the intensity of phloroglucinol staining is a reliable indication of lignification in fiber cells. Lignin composition measurements showed a significant increase in both S- and G-lignin content, as well as an increase in S/G ratio, in the *lbd29-1* mutant (Supplemental Fig. S4B). The T-DNA insertion in *lbd29-1* did not knock-out the expression of the *LBD29* gene but rather interfered with auxin induction, indicating the biological function of LBD29 depends on a precisely regulated expression pattern (Porco et al., 2016). Taken together, our results indicate LBD29 is a negative regulator of SCW development in fiber cells.

### Expression pattern and subcellular localization of LBD29

**Figure 4.**
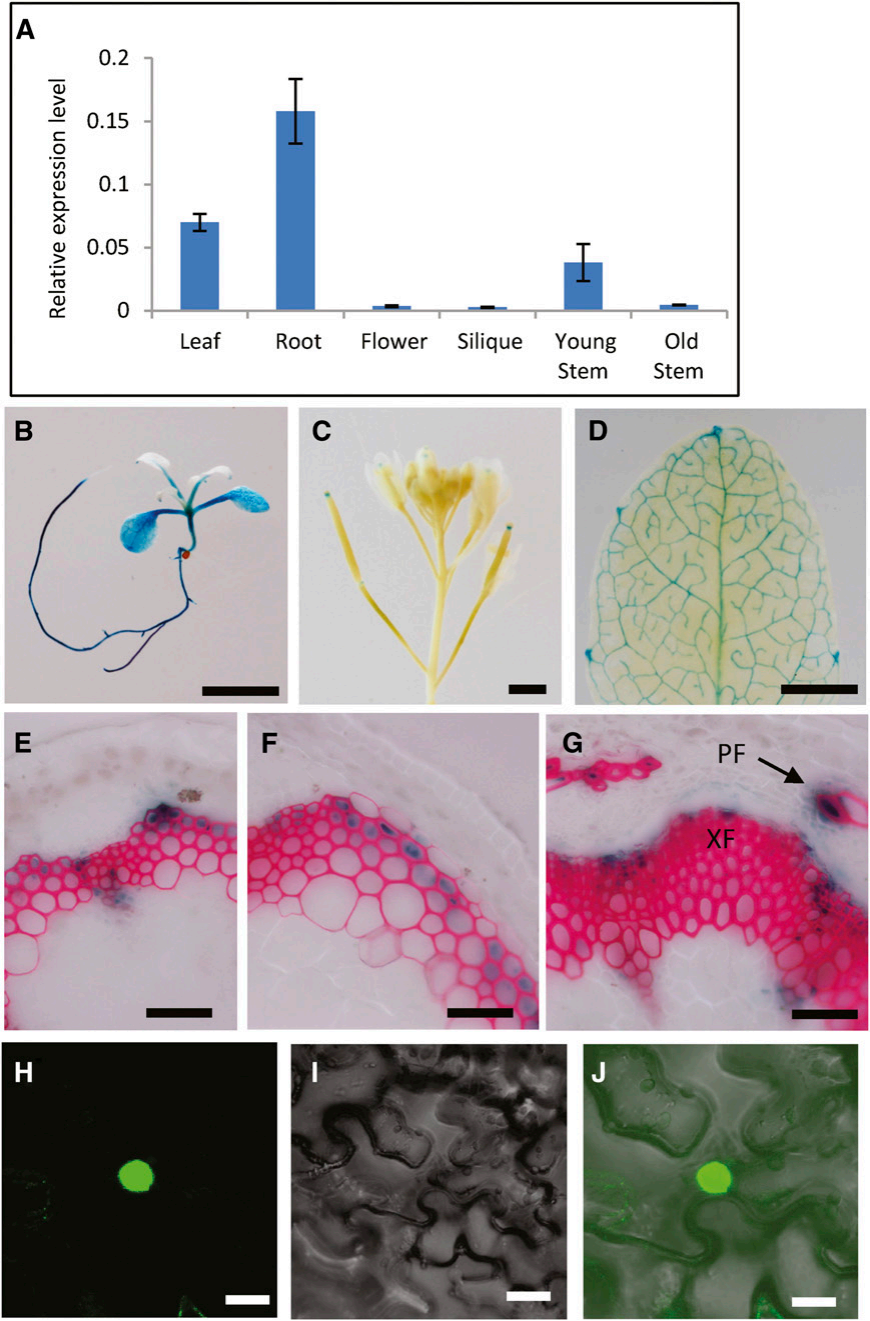
Characterization of the expression pattern of the *LBD29* gene and subcellular localization of the LBD29 protein. A, RT-qPCR analysis of *LBD29* expression in different plant organs relative to the expression of *TUBULIN2*. Young and old stem tissues were collected from the top half and bottom half of the Arabidopsis stem, respectively. B to G, Expression patterns of *LBD29* using a *Pro_LBD29_:GUS* construct. GUS signals were observed in root and cotyledons (B), tips of siliques (C), and vasculatures of leaves (D). Scale bars = 5 mm (B–D). During primary growth, GUS signals were observed in developing fiber cells in fascicular (E) and interfascicular regions (F). During secondary growth, GUS signals were observed in developing secondary xylem fiber (XF) and phloem fiber (PF) cells (G). The cross sections were counterstained with phloroglucinol to show lignified vessel and fiber cells (pink color). Scale bars = 100 μm (E–G). H to J, Characterization of LBD29 localization with LBD29-GFP fusion. Observation of the infiltrated *N. benthamiana* leaves under the GFP fluorescent channel (H), bright light (I), and overlapping images of two channels (J). The GFP signal was localized specifically in the nucleus. Scale bars = 20 μm (H–J).

To better understand the biological function of LBD29, we examined its expression pattern using RT-qPCR analysis as well as a GUS reporter assay. First, expression of *LBD29* was examined in different tissues of the wild-type plants. As shown in Figure 4A, the expression of *LBD29* was high in roots, stems, and leaves but low in flowers and siliques. In addition, the expression of *LBD29* was higher in young stems compared with old stem tissue, suggesting a decreased expression of *LBD29* in tissues with extensive SCW biosynthesis (Fig. 4A). Second, to understand LBD29 expression in detail, we transformed wild-type plants with GUS (β-glucuronidase) reporter driven by a *LBD29* promoter (*Pro_LBD29_:GUS*). In young seedlings, GUS staining was observed in vasculature of roots, cotyledons, and young leaves (Fig. 4B). In mature plants, GUS staining was detected in the tips of siliques and the leaf vasculature (Fig. 4, C and D). Interestingly, high expression level was observed in leaf hydathodes, where auxin accumulates (Fig. 4D). In stem cross sections before secondary growth, GUS staining was observed in young developing fiber cells in both fascicular and interfascicular regions (Fig. 4, E and F). In the mature stems, GUS signals were observed in the developing secondary xylem fibers, phloem fibers, and xylem cells (Fig. 4G). The expression pattern of LBD29 is consistent with its function as a regulator of cell wall development in vascular tissues, and specifically in stem fiber cells in stem tissues.

To examine the subcellular localization of LBD29, we fused LBD29 with GFP under the control of a CaMV 35S promoter and transiently expressed the construct in *Nicotiana benthamiana* leaves. The fusion protein was expressed in the nucleus of the transformed *N. benthamiana* leaves (Fig. 4, H–J). This result is consistent with LBD29 function as a LOB domain transcription factor.

### LBD29 Represses the Expression of NAC Domain Master Regulators

**Figure 5.**
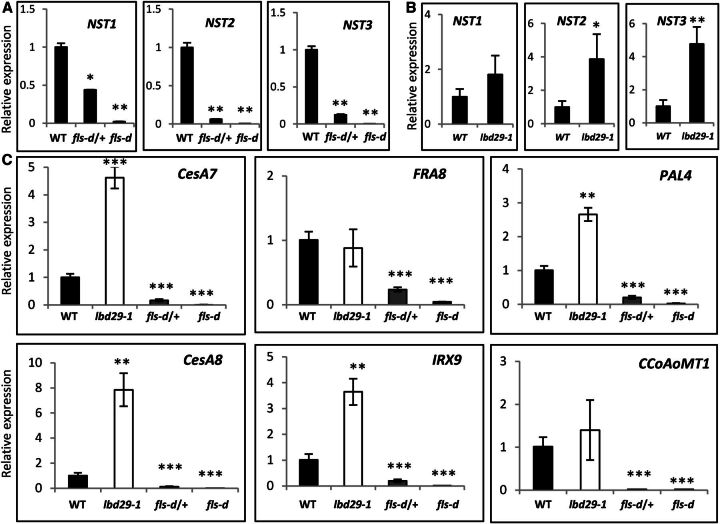
LBD29 negatively regulates the expression of NAC master regulators and wall biosynthesis genes. A, RT-qPCR analysis of the transcripts levels of the master regulatory genes *NST1*, *NST2*, and *NST3* in wild-type (WT) and *fls-d* plants. B, RT-qPCR analysis of transcript levels of *NST1*, *NST2*, and *NST3* in the loss-of-function mutant *lbd29-1*. C, RT-qPCR analysis of transcript levels of wall biosynthesis genes in wild type, *lbd29-1*, and *fls-d* lines. *Significant difference, *P* < 0.05; **significant difference, *P* < 0.01; ***significant difference, *P* < 0.001; Student's *t* test.

The defective SCW in fiber cells of the *fls-d* mutants is very similar to those of *nst1 nst3* double mutant plants (Mitsuda et al., 2007; Zhong et al., 2007). We reasoned the expression of the *NST* genes may be negatively affected in the *fls-d* mutant plants. To test this possibility, we examined the expression of three master regulator genes, *NST1*, *NST2*, and *NST3*, with a real-time RT-qPCR experiment. These results confirmed all three genes were dramatically down-regulated in the heterozygous and homozygous *fls-d* mutant lines (Fig. 5A). These results prompted us to further investigate the expression of these three genes in the LBD29 loss-of-function mutant *lbd29-1*. As shown in Figure 5B, all three *NST* genes were up-regulated in *lbd29-1* mutant compared with wild type. The NAC domain master regulators control SCW development by coordinating the expression of wall biosynthesis genes. To investigate whether SCW biosynthesis was affected in *fls-d* and *lbd29-1* mutants, we examined the expression of genes in cellulose (*CELLULOSE SYNTHASE 7* [*CESA7*] and *CESA8*), hemicellulose (*FRAGILE FIBER 8* [*FRA8*] and *IRREGULAR XYLEM 9* [*IRX9*]), and lignin (*Phenylalanine Ammonia-Lyase 4* [*PAL4*] and *Caffeoyl-coenzyme A O-methyltransferase 1* [*CCoAoMT1*]) biosynthesis. The results indicated all six genes were significantly down-regulated in the *fls-d* mutant lines, and most of them were up-regulated in the *lbd29-1* mutant background (Fig. 5C). These gene expression analyses indicate LBD29 possibly functions as a negative regulator of the three *NST* genes, and as a result, wall biosynthesis genes and SCW development were repressed in fiber cells.

**Figure 6.**
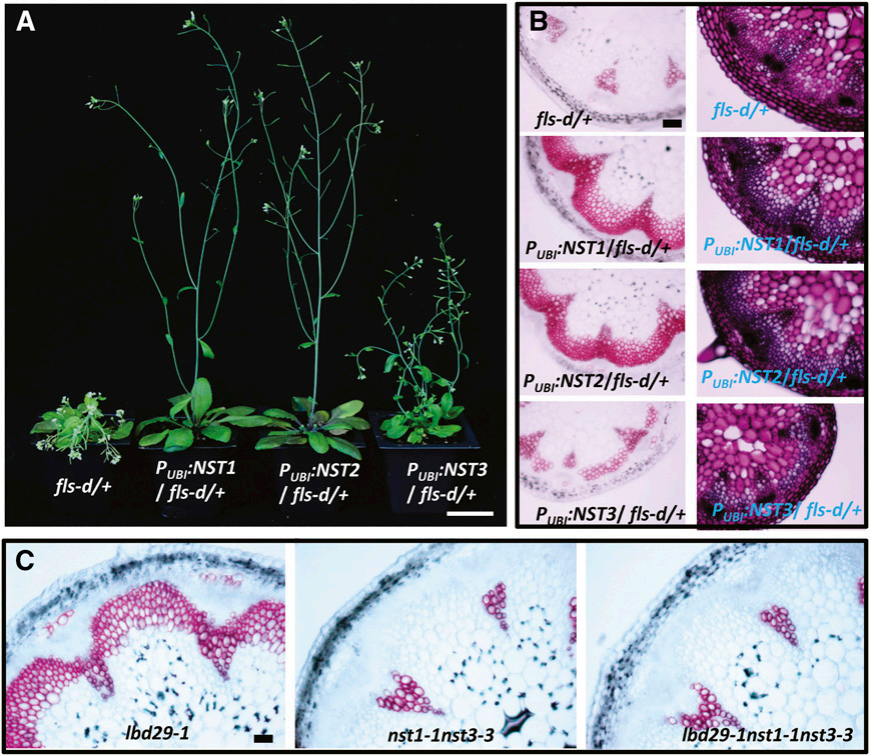
Overexpression of *NST* genes suppresses the *fls-d* mutant phenotypes. A, Plant growth phenotypes of transgenic plants overexpressing *NST1*, *NST2*, or *NST3* in the *fls-d* heterozygous mutant background. Scale bar = 2.5 cm. B, Histochemical characterization of stem cross sections of the transgenic lines with phloroglucinol (dark pink color, left column) and toluidine blue (right column) staining. Scale bar = 100 μm. C, Phloroglucinol staining of stem cross sections of *lbd29-1*, *nst1-1nst3-3*, and *lbd29-1nst1-1nst3-3* plants. Scale bar = 50 μm.

To confirm down-regulation of *NST1*, *NST2* and *NST3* is responsible for the *fls-d* phenotype, we over-expressed these *NST* genes individually in the *fls-d* mutant background. A *UBIQUITIN 10* (*UBQ10*) promoter was used to drive the expression of *NST* genes to avoid possible gene silencing due to the presence of multiple CaMV 35S promoters (Mishiba et al., 2005). Overexpression of *NST1* and *NST2* fully suppressed the *fls-d* mutant growth defects in overall plant growth and the fiberless phenotype in stems (Fig. 6, A and B). On the contrary, overexpression of *NST3* only partially reversed *fls-d* phenotypes (Fig. 6, A and B). These results indicate LBD29 represses SCW development by repressing *NST* genes in stem fiber cells.

Because loss of function of NST1 and NST3 in the *nst1-1nst3-3* double mutant results in a fiberless phenotype, we reasoned mutation of *NST1* and *NST3* in the *lbd29-1* mutant background should repress interfascicular fiber formation if these genes function in a common regulatory pathway. To test this hypothesis, we crossed *lbd29-1* with *nst1-1nst3-3* and generated the *lbd29-1nst1-1nst3-3* triple mutant. As expected, the triple mutant showed no interfascicular fiber formation, similar to the *nst1-1nst3-3* double mutant (Fig. 6C). These results further confirmed the *NST* genes are downstream of *LBD29* in secondary wall biosynthesis in stem fibers.

### Auxin Represses SCW in Fibers through ARF7/ARF19 and LBD29

The *LBD29* gene is one of four LOB domain transcription factor genes (*LBD16*, *LBD18*, *LBD29*, and *LBD30*) known to be induced by auxin, and the induction depends on two auxin response factors, ARF7 and ARF19 (Okushima et al., 2005, 2007). In this research, we also found auxin induced the expression of *LBD29* in stem tissues 6 h after auxin application (Supplemental Fig. S5). Interestingly, we did not observe a significant induction of the expression of *LBD18* and *LBD30* at this time point. The function of LBD29 in repressing SCW biosynthesis suggests auxin and its signaling may play a role in SCW development in fibers.

**Figure 7.**
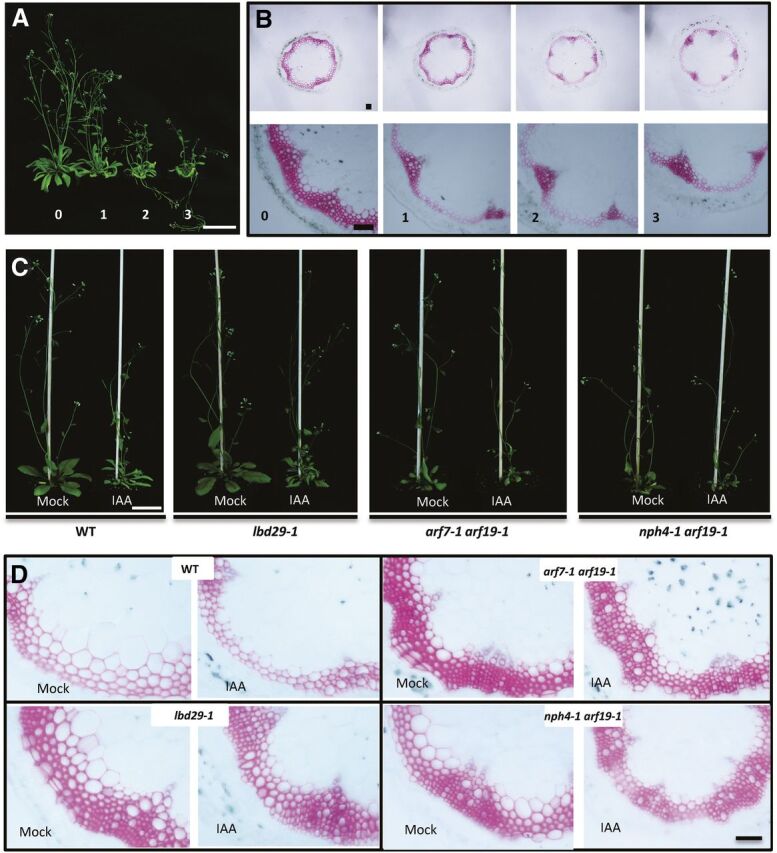
Auxin signaling represses secondary cell wall development through *ARF7/19* and *LBD29* in interfascicular fiber cells. A, Growth phenotypes of wild-type (WT) plants treated with 1 mM of IAA once, twice, or three times per week after bolting. Scale bar = 5 cm. B, Characterization of secondary cell wall development in stem cross sections using phloroglucinol staining (pink color). Scale bar = 100 μm. C, Growth phenotypes of wild type, *lbd29-1, arf7-1 arf19-1*, and *nph4-1 arf19-1* plants treated with 1 mm of IAA once per week. Scale bar = 2.5 cm. D, Characterization of secondary cell wall development using phloroglucinol staining (pink color). Scale bar = 100 μm.

To investigate whether auxin affects fiber wall thickening, we first applied IAA directly on Arabidopsis plants. The effectiveness of direct IAA application on wild-type plants was investigated by spraying 1 mm of IAA once, twice, or three times per week after plant bolting. The development of SCW in fibers was examined after 3 weeks of IAA treatment. The results showed spraying once a week resulted in a significant repression on stem growth and wall thickening in stem fiber cells (Fig. 7, A and B). These results indicated exogenous IAA application can effectively repress fiber wall development in stems in wild-type plants. We then investigated whether mutations in *LBD29* or *ARF7*/*ARF19* influence the effectiveness of auxin treatment on repression of fiber wall development. As shown in Figure 7, plant growth was significantly repressed by IAA treatment in wild-type plants but was not affected in the *lbd29-1* or the two *arf7 arf19* double mutants plants (Fig. 7C). In stem fiber cells, IAA application significantly repressed wall thickening in wild-type plants, but had trivial effects on *lbd29-1* (Fig. 7D). The *arf7-1 arf19-1* and *nph4-1 arf19-1* double mutant lines were also less responsive to auxin treatment compared with wild type but not as dramatic as the *lbd29-1* plants (Fig. 7D). Quantification of the staining intensity further confirmed the mutant lines were less affected by auxin treatment (Supplemental Fig. S6). These results demonstrated auxin has negative effects on fiber wall thickening, and this effect depends on functional auxin signaling involving ARF7/ARF19 and LBD29.

**Figure 8.**
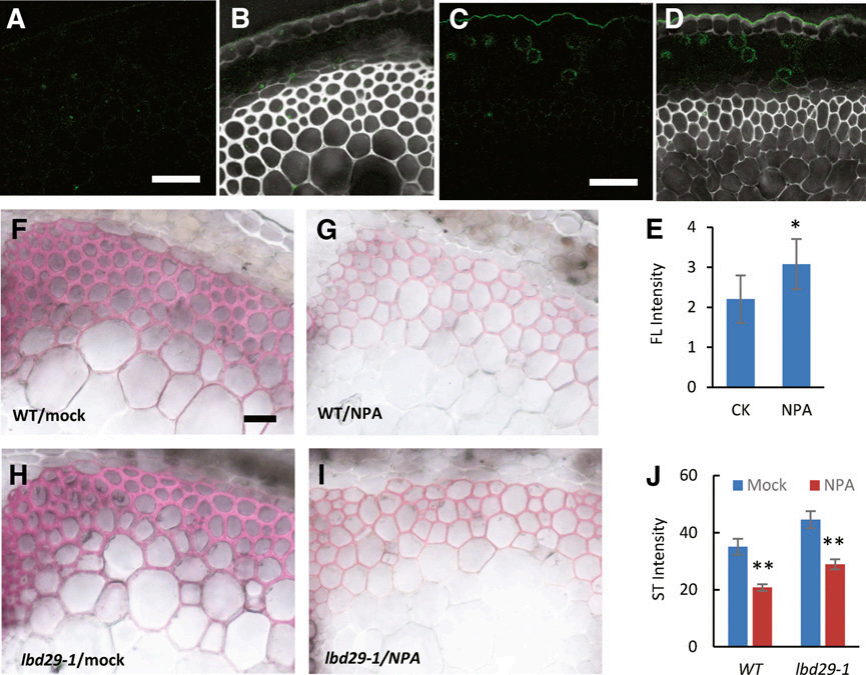
Repression of wall development by elevated auxin levels requires a functional LBD29. A to D, Confocal microscopy images of representative stem cross sections from a DR5:revGFP stable transgenic line (A and C) and overlapping of GFP channel with bright light (B and D). Cross sections were prepared above the mock (A and B) or NPA treatment regions (C and D). Scale bar = 20 μm. E, Measurement of fluorescence intensity in mock- and NPA-treated stem sections. Fluorescence signal intensity was measured using Image J. *Significant difference, *P* < 0.01, Student's *t* test. CK, control; FL, fluorescence. F to I, Histochemical characterization of cross sections in mock-treated (F and H) or NPA-treated plants (G and I). Cross sections were prepared above the mock- or NPA-treated region. Scale bar = 40 μm. J, Quantification of overall staining intensity of stem cross sections in mock- or NPA-treated plants. **Significant difference, *P* < 0.01; Student's *t* test. ST, staining.

Previous studies found application of the auxin transport inhibitor NPA resulted in accumulation of IAA above the application region, which should allow us to manipulate IAA level in a specific stem region (Suer et al., 2011). To confirm the effectiveness of NPA application on IAA level in the stem, we conducted NPA treatment on a reporter line, DR5rev:GFP, to monitor auxin levels by examining the GFP signals (Benková et al., 2003). When compared with the mock-treated control plants (Fig. 8, A and B), increased GFP signals were observed in the cortex and developing interfascicular fiber regions above the NPA application site (Fig. 8, C and D). We further quantified the fluorescence intensity and found NPA treatment increased GFP signal intensity by 28.4%, and the difference was statistically significant (*n* > 12, *P* = 0.023). The experiments were repeated twice with similar results, indicating NPA application is a reliable approach to increase the auxin level above the application zone (Fig. 8E).

To investigate how elevated IAA levels influence SCW development, we applied NPA to stems of wild-type and *lbd29-1* plants and examined the effect 3 d after the treatment. Stem cross sections were prepared above the application region and examined with histochemical analysis. In the wild-type plants, NPA treatment significantly repressed secondary wall thickening in fiber cells (Fig. 8, F, G, and J). In wild-type plants, low staining intensity was observed from NPA treatment samples compared with the mock-treated samples (Supplemental Fig, S7, top two rows). In *lbd29-1* plants, the staining intensity was also lower in NPA treated samples compared with mock treatment, but there was less decrease than in wild-type plants (Supplemental Fig. S7, bottom two rows). There were also noticeable difference in wall thickness under NPA treatment, further supporting the observation that secondary wall formation was less affected in the *lbd29-1* mutant (Supplemental Fig. S7). By measuring the overall staining intensity of the interfascicular fiber regions, we found a 41% decrease in NPA-treated wild-type plants, whereas only a 35% decrease was observed in NPA-treated *lbd29-1* plants (Fig. 8J). These experiments were repeated three times with consistent results, indicating the effect of increased IAA level on secondary wall thickening was less significant in *lbd29-1* mutant plants compared with those of wild-type plants. Intriguingly, SCW formation was also repressed in regions below the NPA treatment, indicating balanced auxin levels are required for proper SCW biosynthesis or deposition (Supplemental Fig. S8).

We further conducted NPA treatment experiments on the *arf7 arf19* double mutant plants. As expected, when compared with wild-type plants, fiber wall thickening was less affected by the NPA treatment in the *arf7 arf19* double mutant lines, *nph4-1 arf19-1* and *arf7-1 arf19-1* (Supplemental Fig. S9, A–F). Taken together, these results indicate increased IAA levels repress SCW development in stem fiber cells, which depends on functional IAA signaling through ARF7/ARF19 and LBD29.

## DISCUSSION

It is well documented that auxin plays a positive role in promoting cambium activity, resulting in enhanced secondary growth (Uggla et al., 1996; Berleth et al., 2000; Donner et al., 2009; Suer et al., 2011). However, the role of auxin in SCW development is still elusive. Previous studies suggest both positive and negative roles of auxin in SCW formation (Zhong and Ye, 2001; Kubo et al., 2005; Agusti et al., 2011; Cecchetti et al., 2013; Ranocha et al., 2013; Didi et al., 2015). The gold standard to establish the function for auxin in SCW biosynthesis is to identify a mutant that is involved in auxin signaling and manifests wall development-related phenotypes. In this study, we report *LBD29* functions in auxin signaling, and gain-of-function of *LBD29* resulted in a fiberless phenotype, whereas loss-of-function of *LBD29* resulted in enhanced wall biosynthesis in fibers. Auxin functions as a negative regulator in SCW biosynthesis under elevated auxin conditions. In particular, we provide both genetic and molecular evidences showing increased auxin levels repress wall development in fibers through a signaling pathway involving ARF7/ARF19 and LBD29.

LBD proteins are transcription factors that regulate important plant developmental processes, such as lateral root formation and callus induction (Okushima et al., 2005, 2007; Fan et al., 2012; Liu et al., 2014). A previous report indicated LBD18 is involved in a positive feedback regulation of VND6/7 in tracheary element development (Soyano et al., 2008). We report here that LBD29 negatively regulates fiber SCW biosynthesis, a process under the regulation of a complex transcriptional network (Taylor-Teeples et al., 2015; Yang and Wang, 2016). In this regulatory network, the NAC master switches NST1/2/3 positively regulate many other downstream transcription factors and wall biosynthesis genes (Wang and Dixon, 2012; Taylor-Teeples et al., 2015; Yang and Wang, 2016). We report here that LBD29 negatively regulates the expression of *NST1, NST2*, and *NST3* in stem fiber cells, consequently repressing the expression of cell wall biosynthesis genes. We have shown *NST1/2/3* and biosynthesis genes of the three major wall components were down-regulated in the *LBD29* gain-of-function *fls-d* mutant line and up-regulated in the *LBD29* loss-of function mutant *lbd29-1* (Fig. 5). These results clearly demonstrated LBD29 functions as a negative regulator of SCW biosynthesis in fibers.

The *nst1-1nst3-3* double mutant shows a strong phenotype with almost no secondary thickening of the fiber cells (Mitsuda et al., 2007; Zhong et al., 2007). The *fls-d* mutant is the only mutant to exhibit a similar phenotype. Gene expression analysis, genetic analyses, and transgenic studies demonstrated that LBD29 represses SCW biosynthesis through negatively regulating NAC domain master regulator genes. Several negative regulators of *NST* genes, such as *MYB4/7/32*, *WRKY12*, and *AtHB15*, have been reported in previous studies (Wang et al., 2010, 2011; Du et al., 2015), but to our knowledge LBD29 is the only negative regulator of *NST* genes in fiber cells. The loss of function mutant *lbd29-1* showed enhanced wall development in interfascicular fiber regions, suggesting that LBD29 has a specific function in fibers. Consistent with its function in fiber wall development, we found that LBD29 is expressed in developing fiber cells in both fascicular and interfascicular regions (Fig. 4, E and F). The negative regulation on secondary wall biosynthesis and its expression pattern also indicate LBD29 may also be responsible for setting the outer boundary of the interfascicular fiber region, thus restricting cells outside of this region from developing secondary walls.

Previously reported *LBD* genes, including *LBD29*, function as transcriptional activators in different developmental processes (Soyano et al., 2008; Feng et al., 2012; Porco et al., 2016; Liu et al., 2019). In this study, the transgenic *ProLBD29:LBD29* plants showed strong overexpression of the *LBD29* gene (Fig. 2). This may be explained by an auxin-induced feedback loop, in which LBD29 activates the auxin influx carrier LAX3 (Porco et al., 2016), which facilitates auxin transport and in turn further induces *LBD29* expression. The function of LBD29 as a negative regulator of NAC domain master switches has two possible explanations. First, LBD29 may function as a direct negative regulator of *NST1/2/3* genes in fiber tissues. It is possible LBD29 is a transcriptional activator but functions as a repressor in specific tissue types (Brackmann et al., 2018). Second, LBD29 functions as an activator of a downstream gene, which itself is a negative regulator that mediates the regulation of *NST* genes and wall biosynthesis. The results from transgenic studies using the dominant repressor LBD29-SRDX fusion support the second model. The transcriptional pathway downstream of *LBD29* in fiber wall biosynthesis is certainly a topic worthy of further investigation.

Consistent with a previous report (Suer et al., 2011), direct application of the auxin transport inhibitor NPA resulted in auxin accumulation above the treatment region (Fig. 7A). We observed repressed SCW biosynthesis on developing stems 3 d after NPA application (Fig. 7B). In addition, application of IAA on wild-type Arabidopsis plants also repressed wall biosynthesis in stem fibers (Fig. 8). These experiments indicate auxin negatively regulates SCW synthesis in fiber cells at increased auxin levels. Surprisingly, in stem sections below the NPA treatment region, SCW biosynthesis was also inhibited, suggesting auxin may also plays a positive role in cell wall biosynthesis under suboptimal auxin conditions (Supplemental Fig. S8). This result is consistent with previous reports of attenuated auxin levels repressing wall development (Zhong and Ye, 1999; Ranocha et al., 2013). Therefore, auxin may play dual roles in SCW formation in fibers.

In *lbd29-1* plants, increased auxin levels also repressed SCW biosynthesis, but the repression was substantially less effective compared with those of the wild-type plants (Figs. 7 and 8). Similarly, repression of wall biosynthesis was also alleviated in the double mutant lines *arf7-1 arf19-1* and *nph4-1 arf19-1* at increased auxin levels (Fig. 8; Supplemental Fig. S9). It is well known that the *LBD29* gene functions downstream of ARF7 and ARF19 in auxin signaling (Okushima et al., 2007). This signaling pathway functions in lateral root formation, callus induction, and adventitious rooting (Okushima et al., 2005, 2007; Fan et al., 2012; Liu et al., 2014). In this study, we found this auxin signaling pathway, i.e. auxin-ARF7/ARF19-LBD29, represses SCW formation in plant stem fibers in response to increased auxin levels. We want to point out that, in stem sections below the NPA treatment zone, SCW formation was repressed (Supplemental Fig. S9), indicating a different molecular mechanism may be involved in the repression of SCW formation under low auxin conditions.

This study reports a specific auxin-signaling pathway repressing the transcriptional master switches, and in turn restricting secondary wall biosynthesis in fibers. Although previous studies have indicated auxin may function as a negative regulator of SCW formation in vitro (Kubo et al., 2005; Didi et al., 2015), this research provides genetic evidence and a molecular mechanism for auxin function in SCW biosynthesis in vivo. The repression function of auxin in SCW formation provides a plausible explanation for the radial distribution of auxin in tree species, i.e. the highest auxin level is found in the cambial zone and auxin levels decrease in SCW-forming cells in wood tissue (Uggla et al., 1996; Tuominen et al., 1997). Further study is needed to fully understand the regulatory mechanisms of wall biosynthesis in response to fluctuations in auxin levels.

## MATERIALS AND METHODS

### Plant Materials and Growth Conditions

The Arabidopsis (*Arabidopsis thaliana*) ecotype Columbia*-*0 (Col-0) was used as wild type. The mutant lines *lbd29-1* (SALK_071133C), *arf7-1 arf19-1* (CS24629), and *arf7-1 nph4-1* (CS24625) were obtained from the Arabidopsis Biological Resource Center (ABRC; The Ohio State University, Columbus). The *fls-d* mutant was identified from a large-scale screening of an activation-tagging mutant population. Plant growth conditions have been described previously (Wang et al., 2010), and the settings in growth chambers were photoperiod, 16-h d/ 8-h night; temperature, 22°C d/ 20°C night; relative humidity 70% to 80%; and light intensity 150 µmol m^−2^ s^−1^.

### Mutant Screening and Phenotypic Characterization

The activation-tagged mutant population was created by transformation of wild-type Arabidopsis plants with activation-tagging vector pSKI015 as described by Weigel et al. (2000). Mutants with defects in vascular development were screened using stem cross sections, which were cut just above the rosette leaves with a Leica RM 2255 microtome. Stem sections were examined using a Nikon Micophot-FX microscope. Micrographs were taken with an INFINITY3 color camera with consistent settings. Mutant plants were further characterized in detail using growth phenotype analysis and cell wall-related histobiochemical staining.

### Determining the Insertion Site of the Activation Tag

The gene responsible for the *fls-d* mutant phenotype was cloned using a Thermal Asymmetric Interlaced PCR (TAIL-PCR) method as described in Liu et al. (1995). Amplified DNA fragments were cut from the agarose gel, purified using a DNA purification kit (Qiagen), and subsequently sent for sequencing. The precise insertion site of the T-DNA containing the activation tag was determined according to the sequencing results. Further genotyping assays and genetic analyses confirmed this single activation tag was responsible for the mutant phenotype. Primers used for genotyping of *fls-d* mutant were pSKI LB3, 5′-TTG​ACC​ATC​ATA​CTC​ATT​GCT​G-3′; FLS-d LP, 5′-ATC​AAA​TAT​CTC​TGC​GGT​TTG​ATG​C-3′; and FLS-d Rp, 5′-CTT​GAC​TAG​CTT​AGT​CAT​ATG​TTC​AC-3′.

### Constructs and Plant Transformation

To make overexpression constructs, the coding sequences of *At3g58180* (an Armadillo [ARM] repeat protein), *At3g58190* (*LBD29*), At3g58193 (a small nucleolar RNA [SnoRNA]), *At3g58200* (a TRAF-like protein), *At3g58210* (a TRAF-like protein) were cloned using high-fidelity polymerase and inserted into the TOPO-d vector. Primers for cloning these genes were provided in Supplemental Table S1. The overexpression binary vector had a hygromycin resistance gene (pBI-Hyg; Lee et al., 2003). All constructs were confirmed by sequencing and subcloned to destination vector pK2GW7 by LR reaction. To make the Pro_LBD29_:GUS reporter line, a 1.5 kb DNA fragment up-stream of the start codon was amplified and inserted into the TOPO-d vector. The primers used for cloning the promoter sequence of *LBD29* were pLBD29F 5′-CAC​CGG​TTA​AGA​TTA​TAG​TTC​TCA​GTA​TTG​CA-3′ and pLBD29R 5′-GAT​GAT​GAT​GGT​GTT​GTG​ACG-3′. After sequencing, the promoter sequence was subcloned into pBGWFS7 using the LR reaction. All constructs in binary vectors were transformed into *Agrobacterium tumefaciens* strain GV3101 for plant transformation.

The floral dipping method was used for Arabidopsis transformation (Clough and Bent, 1998). Seeds collected from the transformed Arabidopsis plants were plated on half-strength Murashige and Skoog (MS) medium supplied with appropriate antibiotics. Resistant plants that survived on the selection plate were transferred to soil for further analysis.

### GUS Staining

Staining of GUS reporter lines was performed as described in Wang et al. (2010). To stain the stem tissues, cross sections were cut at 100-120 µm and submerged into the staining buffer (2 mM X-Gluc [5-bromo-4-chloro-3-indolyl glucuronide]; 50 mM Na_2_HPO_4_, pH 7.0; 5 mm potassium ferricyanide/ferrocyanide; and 0.06% [v/v] Triton X-100). Samples were infiltrated under vacuum for 10 min and then incubated at 37°C overnight. Staining buffer was replaced with 70% ethanol to clear the tissue.

### Gene Expression Analysis

To analyze gene expression, plant tissues were collected from stems of 35-d-old plants or whole seedlings, flash frozen in liquid nitrogen, and stored in a −80°C freezer. Total RNAs were isolated using a RNA isolation kit (Qiagen). RNA samples were treated with RNase-free DNase (Qiagen) to eliminate contamination from genomic DNA. Three micrograms of total RNA was reverse transcribed using the Superscript III RT kit (Invitrogen) in a 20 µL reaction system. The complementary DNA was diluted 50 times and used as the templates for RT-PCR or RT-qPCR as previously described (Wang et al., 2010). Primers used for RT-qPCR are listed in Supplemental Table S1.

### NPA Treatment

*N*-1-naphthylphthalamidic acid (NPA, SUPELCO) was first dissolved in dimethyl sulfoxide and then mixed with lanolin (Sigma-Aldrich)/paraffin at a final concentration of 1% (w/w). This treatment has previously been used to treat Arabidopsis stems (Suer et al., 2011). In brief, NPA was applied to the first internode of 5- to 7-cm-tall plants at a distance of at least 1.5 cm from the stem base. A ring of lanolin was applied around the stem, resulting in a treatment ring 4 to 5 mm wide. After 3 d of incubation, stem segments were harvested and analyzed histologically.

### Auxin Treatment

To prepare the IAA solution, we first solubilized IAA in 100% ethanol and then diluted in water to a final concentration of 1 mM (final 0.1% ethanol). The control solution consisted of water with 0.1% ethanol. Individual plants at approximately the same developmental stage were selected for treatment starting from bolting (about 30-d postgermination). Plants were treated by evenly spraying with IAA or control solution every 7 d (1, 8, 15 d after bolting). Arabidopsis plants (48-d-old) were imaged 3 d after the last treatment. Plant stems were prepared for biochemical staining and microscopy.

### Analysis of Histological Staining and Fluorescence Signal Intensity

To analyze the fluorescence signal of DR5:revGFP in mock- or NPA-treated stem cross sections, we used ImageJ software. The interfascicular cambium and cortex regions were selected as the regions of interest using the polygon selection tool. Fluorescence intensity quantification was performed using the Analyze-Measure tool of ImageJ software (Jensen, 2013). At least 12 independent images were analyzed from each treatment. The mean intensity of each measurement was used to calculate the overall intensity of each sample. A two-tailed Student's *t* test was used for statistical analysis.

ImageJ software was also used to quantify the intensity of phloroglucinol stained stem cross sections. The images were first split into single channels using the Image-Type-HSB (Hue, Saturation, Brightness) stack function. The S (saturation) channel represented the intensity of phloroglucinol staining. Staining intensity of the interfascicular fiber regions was then measured similarly to a fluorescence intensity measurement. The intensity values across cells were obtained using the Analyze-Plot profile function. The plot gave the intensity values along the line drawn across interfascicular fiber cells. Each peak represented the intensity of each cell wall along the line.

### Measurement of Lignin Content and Composition

Samples of 30-d-old stem from wild-type and *lbd29-1* plants were ground with a freeze mill and extracted sequentially with methanol, chloroform/methanol (2:1, v/v), methanol and water three times each, and then lyophilized. Lignin content was determined by the acetyl bromide method, and lignin composition was determined by the thioacidolysis method (Du et al., 2015). Lignin content and composition were measured using three biological replicates, each with technical replicates.

### Accession Numbers

Sequence data from this article can be found in the EMBL/GenBank data libraries under the following accession numbers: LBD29 (At3g58190), NST1 (At2g46770), NST2 (At3g61910), NST3 (At1g32770), ARM repeat protein (At3g58180), SnoRNA (At3g58193), TRAF like protein 1 (At3g58200), TRAF like protein 2 (At3g58210), CESA7 (At5g17420), CESA8 (At4g18780), FRA8 (At5g22940), IRX9 (At2g37090), PAL4 (At3g10340), and CCoAOMT (At4g34050). Mutants used in this article can be obtained from ABRC under the following accession numbers: *lbd29-1* (SALK_071133C), *arf7-1 arf19-1* (CS24629), *arf7-1 nph4-1* (CS24625), *nst1-1* (SALK_120377), and *nst3-3* (SALK_015495).

### Supplemental Data

The following supplemental materials are available.

**Supplemental Figure S1.** Expression analysis and transgenic characterization of genes close to the activation-tag insertion locus.
**Supplemental Figure S2.** Overexpression of *LBD29* using a CaMV 35S promoter repressed fiber wall development.
**Supplemental Figure S3.** Overexpression of a dominant negative construct driven by a CaMV 35S promoter resulted in ectopic secondary cell wall development.
**Supplemental Figure S4.** Measurement of total lignin content and composition.
**Supplemental Figure S5.** Expression of *LBD29* is induced by IAA treatment in stem tissue.
**Supplemental Figure S6.** Measurement of staining intensity after IAA treatment in different lines.
**Supplemental Figure S7.** Measurement of wall staining intensity using the Plot Profile function in Image J software.
**Supplemental Figure S8.** Fiber wall development is repressed in regions below NPA treatment.
**Supplemental Figure S9.** Repression of wall development by high auxin levels in regions above NPA treatment requires functional IAA signaling involving ARF7 and ARF19.
**Supplemental Table S1.** Primers used for cloning and RT-qPCR analysis.

## Supplementary Material

Supplementary Data

Supplementary Data
