# Supplementary material for: LBD29-Involved Auxin Signaling Represses NAC Master Regulators and Fiber Wall Biosynthesis
Source: Plant Physiol. 2019 Aug 3;181(2):595–608. doi: 10.1104/pp.19.00148 (PMC6776862; doi:10.1104/pp.19.00148)
Supplement: Supplementary Data [file plphys_v181_2_595_s1.zip › PP2019-RA-00148R2_Supplemental_Material.pdf]

Fig. S1

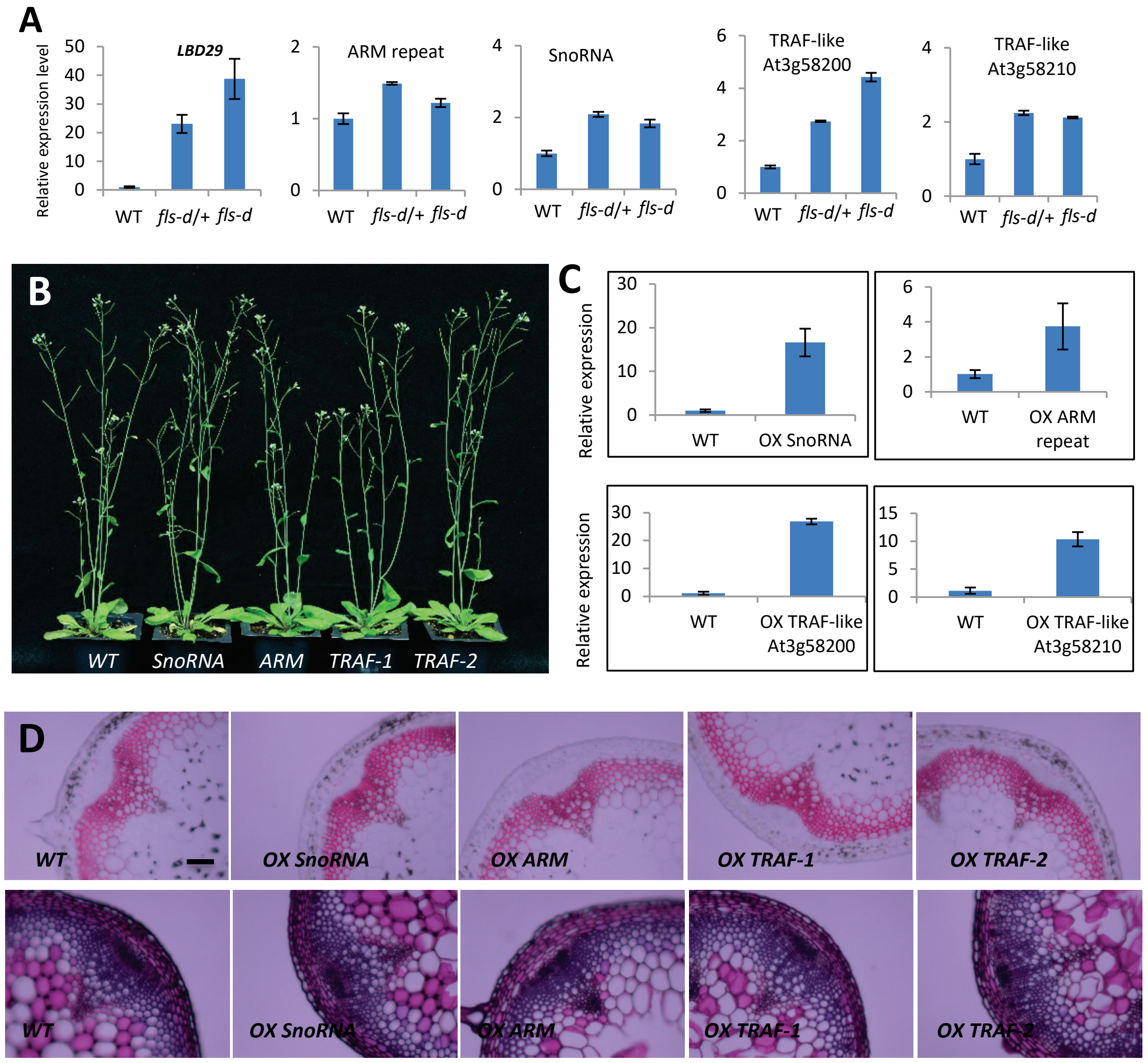


**Supplementary Figure S1** Expression analysis and transgenic characterization of genes close to the activation-tag insertion locus. A, Real-time RT PCR analysis of five genes in a 10 kb range from the activation-tag insertion locus. These genes are *LBD29* (At3g58190), *SnoRNA* (At3g58193), a *ARM* repeat gene (At3g58180), TRAF like 1(At3g58200) and TRAF like 2 (At3g58210). B, Plant growth phenotypes of wild type and transgenic lines overexpressing *SnoRNA*, *ARM repeat*, *TRAF-1* and *TRAF-2*. No obvious differences in growth phenotype were observed in these transgenic lines. C, Real time PCR analysis of the transgenic lines. Overexpression of the corresponding genes was confirmed in the transgenic lines. D, Histochemical characterization of wild type and the transgenic overexpression lines. Phloroglucinol staining (upper panels) and toluidine blue staining (lower panels) showed normal development in vascular and interfascicular regions. Scale bar=100µm.

# Fig. S2

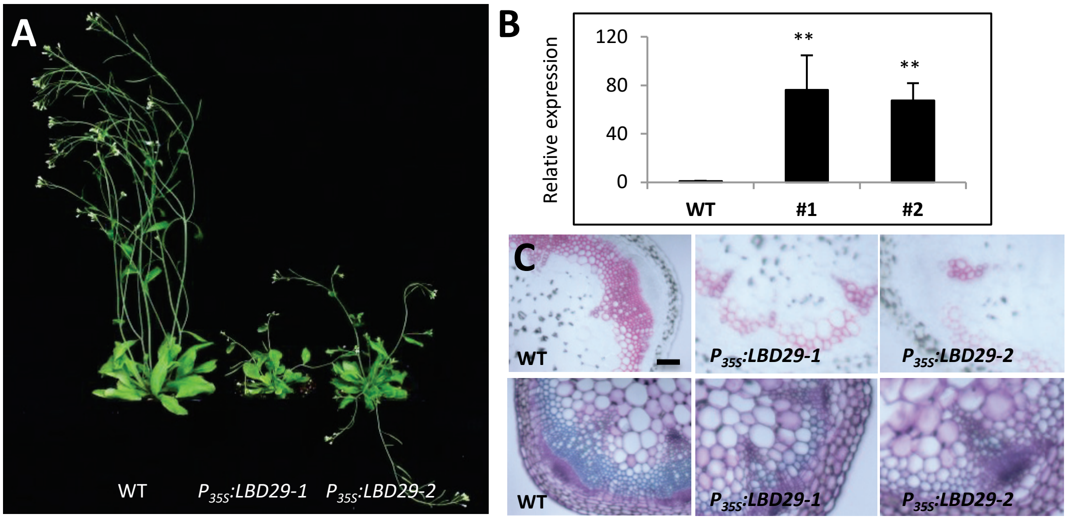


**Supplementary Figure S2** Overexpression of *LBD29* using a CaMV 35S promoter repressed fiber wall development. A, Growth phenotypes of wild type (WT) and two overexpression lines in which *LBD29* driven by a CaMV35S promoter (note the pendulous stems of the overexpression lines). B, Real-time RT-PCR analysis of *LBD29* expression showing overexpression in the transgenic lines. ** denotes strong significant difference, Student t-test, $p<0.01$. C, Histochemical characterization of stem cross sections of two *LBD29* OX lines showing reduced cell wall thickening in fibers. Scale bar=100μm.

# Fig. S3

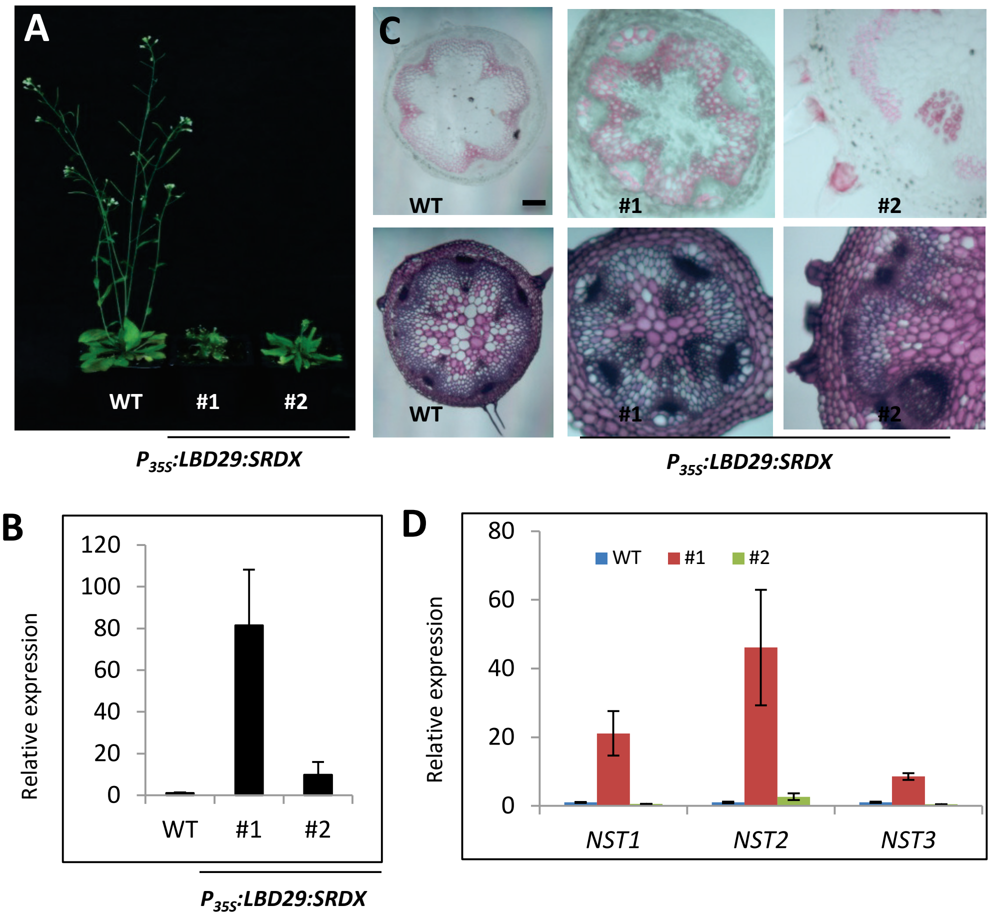


**Supplementary Figure S3.** Overexpression of a dominant negative construct driven by a *CaMV 35S* promoter resulted in ectopic secondary cell wall development. A, Plant growth phenotypes of wild type and two representative transgenic plants expressing a $Pro_{CaMV35S}$*:LBD29-SRDX* construct. Transgenic plants are extremely dwarf compared to wild type plants. B, Real-time RT-PCR analysis showing expression of *LBD29-SRDX* compared to wild type. C, Histochemical characterization of stem cross sections with phloroglucinol staining (upper panel) and toluidine blue staining (lower panel). Ectopic secondary wall formation was observed in phloem fiber cells and epidermal cells in the transgenic lines. Scale bar=200 µm. D, Real-time PCR analysis of *NST1*, *NST2* and *NST3* showing increased expression of these three master regulators in the transgenic lines.

Fig. S4

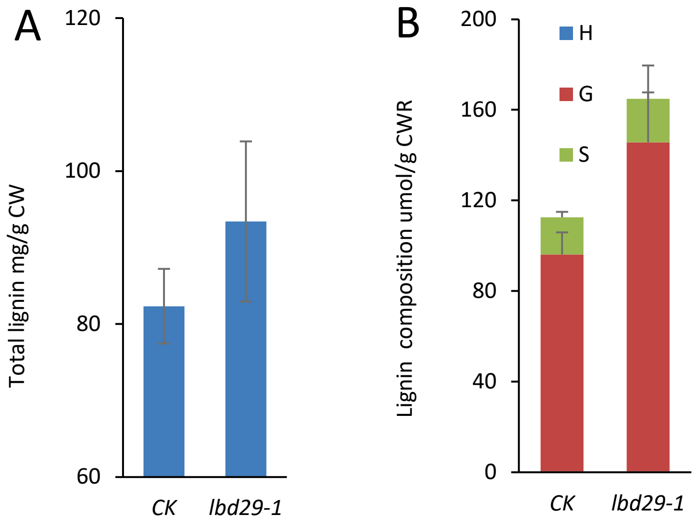


**Supplementary Figure S4.** Measurement of total lignin and lignin composition. A, measurement of total lignin. Total lignin was measured using the acetyl bromide method. B, lignin composition measurement with thioacidolysis.

Fig. S5

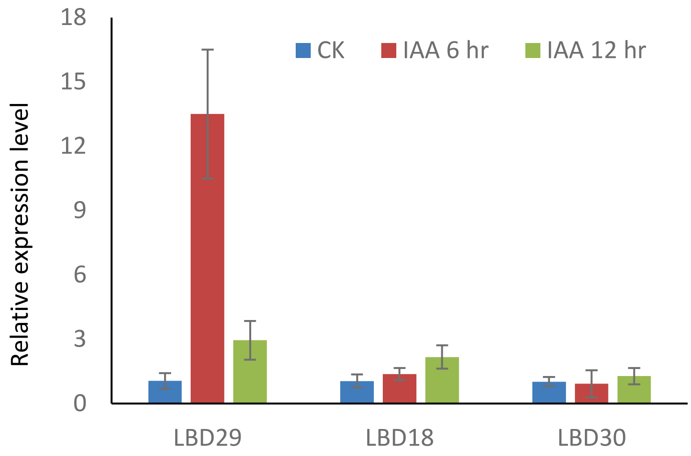


**Supplementary Figure S5.** Expression of *LBD29* is induced by IAA treatment in stem tissue.

Fig. S6

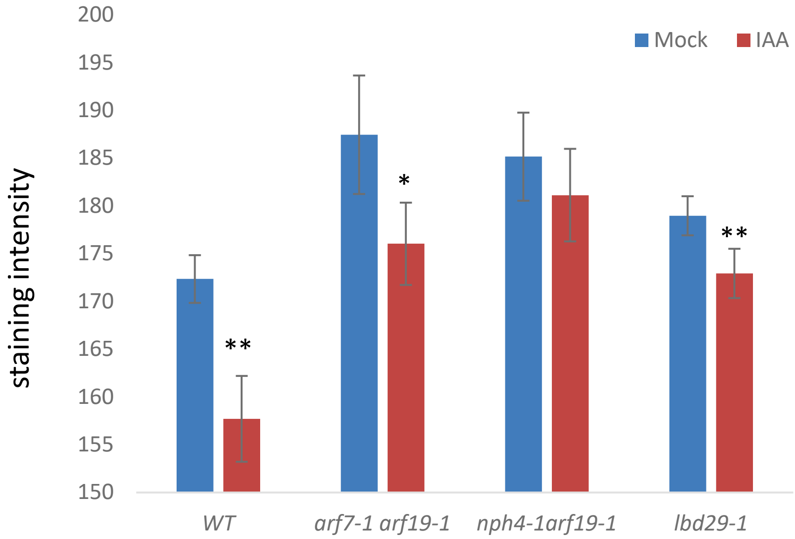


**Supplementary Figure S6.** Measurement of staining intensity after IAA treatment in different lines.

# Fig. S7

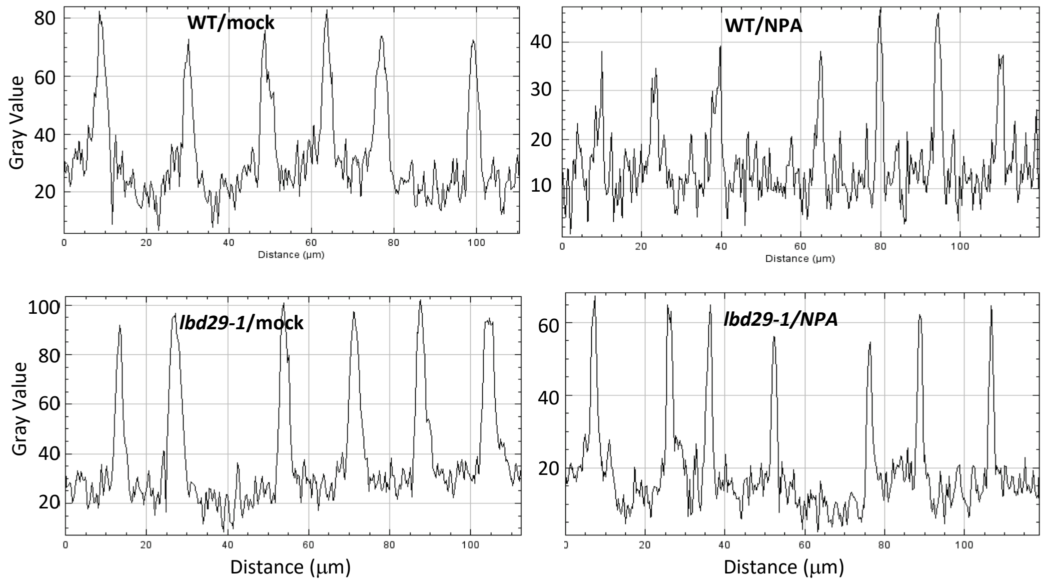


**Supplementary Figure S7.** Measurement of wall staining intensity using the Plot Profile function in Image J software. Staining intensity was measured across 6-7 cells, noting differences of staining intensity after NPA treatment in wild type and *lbd29-1* mutant plants.

# Fig. S8

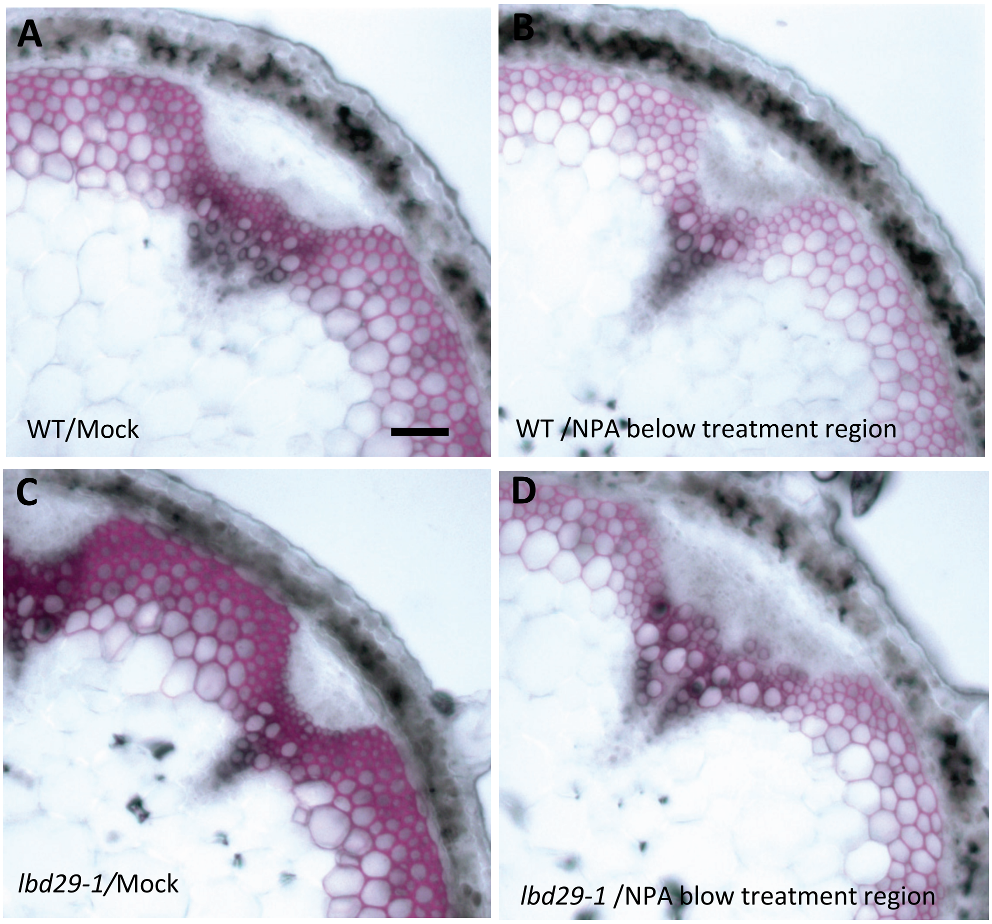


**Supplementary Figure S8.** Fiber wall development is repressed in regions below NPA treatment. A-D, Histochemical characterization of cross sections in mock treated (A) and (C), or NPA treated plants (B) and (D). Cross sections were prepared below the mock or NPA treated region, noting that NPA treatment repressed fiber wall thickening in both wild type and *lbd29-1* mutant plants. Scale bar=100 μm.

Fig. S9

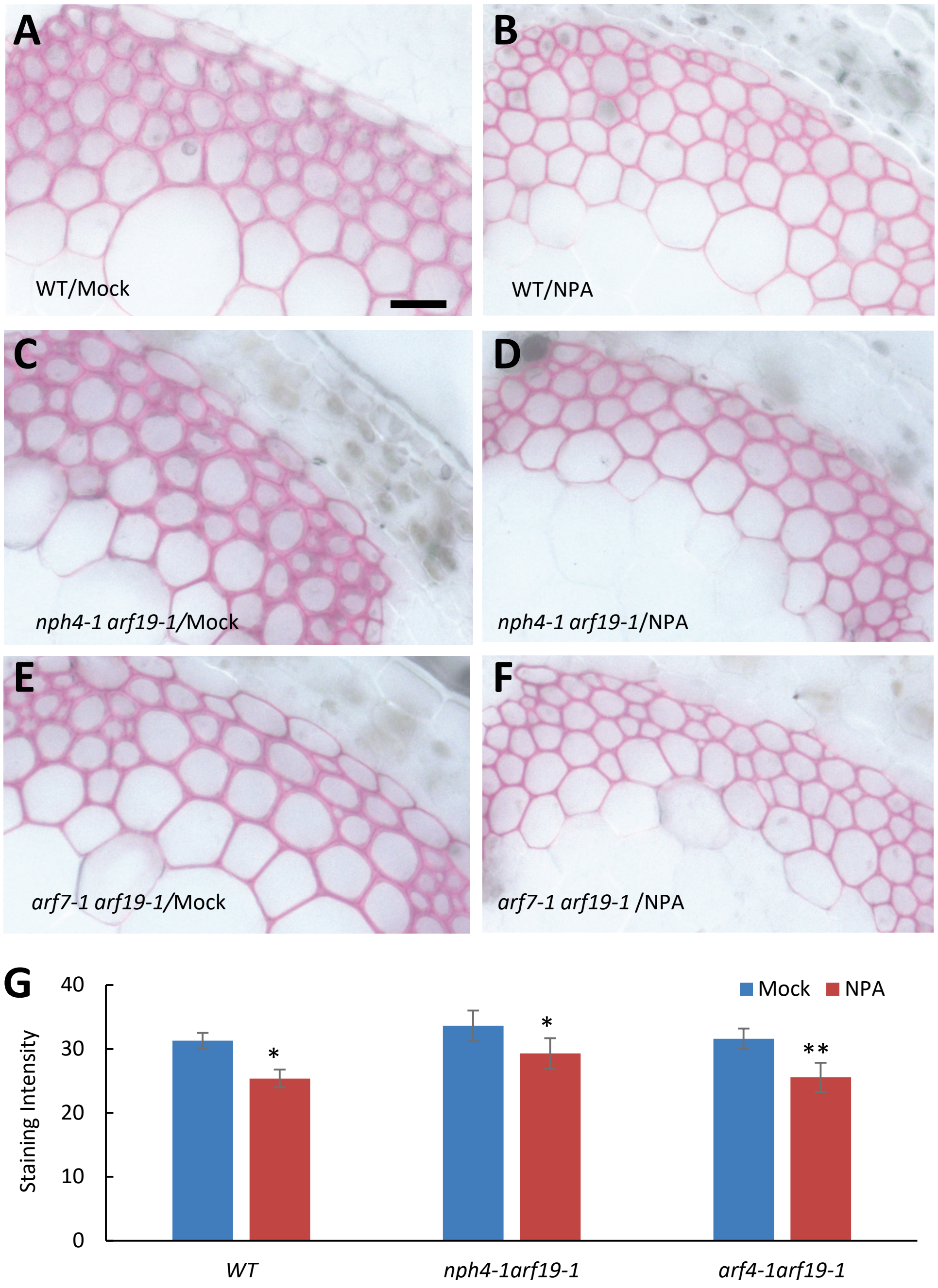


**Supplementary Figure S9.** Repression of wall development by high auxin levels in regions above NPA treatment requires a functional IAA signaling involving ARF7 and ARF19. A-F,Histochemical characterization of cross sections in mock treated (A), (C) and (E), or NPA treated plants (B), (D) and (F). Cross sections were prepared above the mock or NPA treated region, noting that NPA treatment repressed fiber wall thickening, but the repression is less significant in the *nph4-1 arf19-1* and *arf7-1arf19-1* double mutant plants. Scale bar=40 µm. G, Staining intensity quantification of stem cross-sections of mock and NPA treated plants. * denotes P<0.05, ** denotes P<0.01 as determined by two tails Student T-test.

**Supplementary Table 1.** Primers used for cloning and RT-qPCR analysis.

| Primer name | Primer sequence |
|---|---|
| LBD29-OXFw | 5’-CTAGTCTAGAATGACTAGTTCCAGCTCTAGCTCT-3’ |
| LBD29-OXRe | 5’-TCCCCCCGGGTCACGAGAAGGAGATGTAGCCAA-3’ |
| LBD29-SRDX-Re | TCCCCCCGGGTCAAGCAAACCCTAAACGCAACTCCAAGTCTAAGTCAAGCGAGA AGGAGATGTAGCCAA-3’ |
| ARM-At3g58180 Fw | CTAGTCTAGAATGGAATCTAATGGATCAGTTTCATC |
| ARM-At3g58180 Re | TCCCCCCGGGTTAGTGAACAAGCGGGTCTTG |
| TRAF-AT3G58200 Fw | CTAGTCTAGAATGGAGAAAGAAGCTGATAACAAG |
| TRAF-AT3G58200 Re | TCCCCCCGGGTCAAAGAACATCATCCAACGTTAG |
| TRAF-AT3G58210 Fw | CTAGTCTAGAATGGGAAATCTCGTTGATAACAAGT |
| TRAF-AT3G58210 Re | TCCCCCCGGGTTAAATAACATCATCCAATGTTAGAGGAG |
| SnoRNA- At3g58193 Fw | CTAGTCTAGAAGATATGATGATGGAATTAGATTTCC |
| SnoRNA- At3g58193 Re | TCCCCCCGGGCAGAGTTAAAGGGAAGAGAATGA |
| NST1-TOPO Fw | CACCATGATGTCAAAATCTATGAGCATATC |
| NST1-TOPO Re | TTATCCACTACCATTCGACAC |
| NST2-TOPO Fw | CACCATGAACATATCAGTAAACGGACAG |
| NST2-TOPO Re | TTATCCACTACCGTTCAACAAG |
| NST3-TOTO Fw | CACCATGGCTGATAATAAGGTCAATCT |
| NST3-TOPO Re | TCATACAGATAAATGAAGAAGTGG |
| AT3G58200-Realtime Fw | GAGGAATCTGAAGAGGCAACA |
| AT3G58200-Realtime Re | CCTGGGAAGGAAGAACATGAA |
| AT3G58210-Realtime Fw | AAGTGGCGTCTTCTTGTCTATC |
| AT3G58210-Realtime Re | CATCCAGGTGGCAATGATCT |
| SnoRNA-Realtime Fw | TGAAGTGATGATTGAACTTTGTTTC |
| SnoRNA-Realtime Re | GATCAGATAGAGCTAATACGTATG |
| LBD29-Realtime Fw | TGGTTTACCAAACTGGAAGTTC |
| LBD29-Realtime Re | CTGATTGAAGCTCTTTGAGATG |
| NST1-Realtime Fw | GTAAGCTCTCCCGACACTAATC |
| NST1-Realtime Re | CCGCCCAGCTAGTAACAAA |
| NST2-Realtime Fw | CCCTAACCTCGAATGCCATAA |
| NST2-Realtime Re | TGATGAAGCTGGGATCCATAAC |
| NST3-Realtime Fw | CCCAAGTCTTGAGACACCTAAAT |
| NST3-Realtime Re | CCGGTCTTTCGGTTATCTTCTC |
| CesA7-Realtime Fw | TTGTTGCAGGCATCTCAGATG |
| CesA7-Realtime Re | GCAGTTGATGCCACACTTGGA |
| CesA8-Realtime Fw | TGAGCTTTACATTGTCAAATG |
| CesA8-Realtime Re | GCAATCGATCAAAAGACAGTT |
| FRA8-Realtime Fw | GACTTGTTGAATCGGTGGCTC |
| FRA8-Realtime Re | GAAAGAGTTTGACCTTCTAAC |
| IRX9-Realtime Fw | TCCTCCCCAACCTTTTGAAA |
| IRX9-Realtime Re | TTGGGAATTGATCAAGGTTCTATTG |
| PAL4-Realtime Fw | CCTCCGGTGACCTTGTTCCT |
| PAL4-Realtime Re | AGTTGGGACGGCCAGTGA |
| CCoAOMT-Realtime Fw | ACAAGAAACTCTCGGCTGATCAG |
| CCoAOMT-Realtime Re | TCGCCAAGCGCAGCTT |
| UBQ5-Realtime Fw | CTGCATTTCTATTTGGGAATTTTGTAA |
| UBQ5-Realtime Re | ATCCAGAACGAAAGATGTTCAACATA |
| ACT2-Realtime Fw | GCCATCCAAGCTGTTCTCTCC |
| ACT2-Realtime Re | TTCTCGATGGAAGAGCTGGT |
| | |
